# Renal toxicity and biokinetics models after repeated uranium instillation

**DOI:** 10.1038/s41598-023-31073-1

**Published:** 2023-03-13

**Authors:** Laurie De Castro, Annabelle Manoury, Olivier Claude, Bastien Simoneau, Virginie Monceau, David Suhard, Christelle Elie, Victor Magneron, Laurence Roy, Céline Bouvier-Capely, Chrystelle Ibanez, Estelle Davesne, Yann Guéguen

**Affiliations:** 1 Institut de Radioprotection et de Sûreté Nucléaire (IRSN), PSE-SANTE, SESANE, B.P. N°17, 92262 Fontenay-Aux-Roses Cedex, France; 2 Institut de Radioprotection et de Sûreté Nucléaire (IRSN), PSE-SANTE, SDOS, Fontenay-aux-Roses, France

**Keywords:** Kidney, RNA, Respiratory system models, Ultrasound, Metals, Risk factors, Immunohistochemistry

## Abstract

During nuclear fuel processing, workers can potentially be exposed to repeated inhalations of uranium compounds. Uranium nephrotoxicity is well documented after acute uranium intake, but it is controversial after long-term or protracted exposure. This study aims to analyze the nephrotoxicity threshold after repeated uranium exposure through upper airways and to investigate the resulting uranium biokinetics in comparison to reference models. Mice (C57BL/6J) were exposed to uranyl nitrate (0.03–3 mg/kg/day) via intranasal instillation four times a week for two weeks. Concentrations of uranium in urines and tissues were measured at regular time points (from day 1 to 91 post-exposure). At each exposure level, the amount of uranium retained in organs/tissues (kidney, lung, bone, nasal compartment, carcass) and excreta (urine, feces) reflected the two consecutive weeks of instillation except for renal uranium retention for the highest uranium dose. Nephrotoxicity biomarkers, KIM-1, clusterin and osteopontin, are induced from day 4 to day 21 and associated with changes in renal function (arterial fluxes) measured using non-invasive functional imaging (Doppler-ultrasonography) and confirmed by renal histopathological analysis. These results suggest that specific biokinetic models should be developed to consider altered uranium excretion and retention in kidney due to nephrotoxicity. The threshold is between 0.25 and 1 mg/kg/day after repeated exposure to uranium via upper airways.

## Introduction

The biodistribution and health effects of uranium depend on the speciation of uranium compounds, the isotopic composition, duration and route of exposure^1–3^. Among known nephrotoxic agents, uranium is a radio element with known chemical (as a heavy metal) and radiological (α-emitting radionuclide) toxicities, which accumulates preferentially in the kidneys and more specifically in proximal convoluted tubules causing kidney toxicity at high dose. Abundant existing literature, including our previous studies, showed that acute uranium exposure induces renal tubular damage associated with kidney function impairment in animals^4–8^ and humans^9–11^. But, the level of uranium induced nephrotoxicity is controversial after chronic or protracted exposure, particularly after inhalation^12–15^. Repeated inhalation of uranium compounds can occur in several situations including nuclear fuel processing for workers, military activities using depleted uranium munitions, and non-war situations such as crashed aircraft^16,17^.

The kidney is a key organ in maintaining ion and fluid homeostasis, eliminating metabolic degradation products, detoxification by elimination of xenobiotics, and the biosynthesis of some hormones. Renal dysfunction can therefore lead to severe disorders that cause the individual's general health status to deteriorate due to renal failure that can lead to death. Most damage caused by xeno-induced renal disease affects the proximal tubular epithelial cells (PTECs)^18^, an area of maximum transport, secretory, and metabolic activity. They are the most sensitive target cells affected by uranium toxicity as reported by experimental studies showing oxidative stress, inflammation, DNA damage, and induced cell death. This alteration to the molecular pathway induces histological and tubular biomarkers^2,15,19–21^ including the transmembrane protein Kidney Injury molecule-1 (KIM-1), a sensitive biomarker of proximal convoluted tubule injury^4,22^.

It is essential to correlate knowledge of how a toxic element like uranium is retained and excreted with biological effects. A biokinetic model after occupational uranium intake has recently been updated by the International Commission on Radiological Protection (ICRP)^23^. However most experimental data either consider acute inhalation, chronic ingestion via drinking water, or wound contamination^24–26^. Repeated acute intake is assumed in order to evaluate uranium kidney concentration after protracted inhalation, but few experimental data are available to support this assumption^27,28^. The procedure for the single or repeated intra-nasal instillation of chemicals in rodents is well described in the literature for modeling upper airway exposure^29–31^, including uranium exposure^32^.

This study aims to define the dose–response relationship of kidney impairment due to uranium induced nephrotoxicity in an exposure model representative of occupational or military exposure to uranium via inhalation. The objective of this work is therefore to verify (1) if the uranium biokinetic model developed from data gathered after acute inhalation is consistent with data obtained after repeated exposure of animals via intra-nasal instillations, (2) if nephrotoxicity modifies uranium retention and excretion (3) if the nephrotoxicity threshold can be predicted by the models.

C57BL/6J mice were exposed to the repeated instillation of uranium in a dose–response study (0.25, 1 or 3 mg/kg/day) and groups of animals were euthanized at regular time points (2, 4, 7, 11, 21 and 91 days after the first treatment). Uranium biokinetics were monitored in the kidneys, lungs, bones, urine, feces, nasal compartments, gastrointestinal tract, and the remaining carcass. Nephrotoxicity was evaluated by (i) high resolution ultrasonography (US) with Doppler mode to identify morphological and functional changes, (ii) renal anatomopathological scoring of lesions induced by uranium exposure, (iii) biomarker measurements in urines or renal tissues by KIM-1 ELISA, immunostaining or gene expression RT-PCR assay.

## Results

### Uranium biokinetics

**Figure 1:**
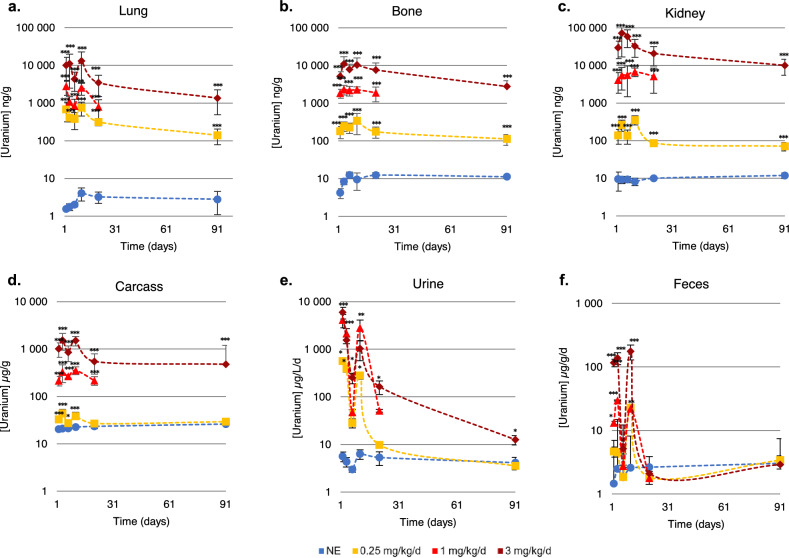
Uranium content in lungs, bones, kidneys, urine, feces and remaining carcass after repeated intranasal instillation (0.25–3 mg/kg/day) from Day 2 to Day 91 after the first instillation. Uranium concentration was evaluated in the tissue by ICP-MS and expressed in ng/g tissue wet weight. The values are expressed as mean ± SD. *NE* non-exposed. *p < 0.05/**p < 0.01/***p < 0.001, comparison with unexposed animals, Holm-Sidak test.

**Figure 2:**
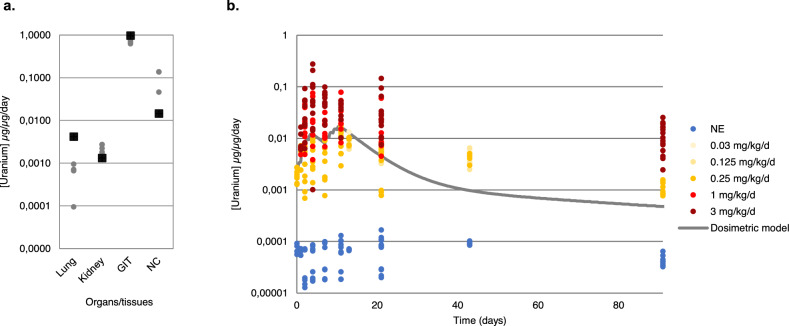
Uranium tissue content of mice exposed to intranasal instillation. (**a**) Uranium retention in lungs, kidneys, gastrointestinal tract, and nasal compartments 1 h after the first instillation to uranium of 0.25 mg/kg. Each point represents an independent animal, and the square represents the SAAM II (Simulation, analysis, and modeling software for tracer and pharmacokinetic studies) biodosimetric model, n = 4. *GIT* gastro-intestinal tractus, *NC* nasal compartment. (**b**) Biodosimetric model of uranium content in the kidneys after repeated intranasal instillations (0.03–3 mg/kg/day) according to the quantity of uranium administered to mice. Each point represents an independent animal; n = 4–12 for each time and dose.

Specific groups of mice were used to evaluate the biokinetics of uranium on days 2, 4, 7, 11, 21 and 91 after the first treatment day. The renal, lung, bone femur and carcass retention of uranium and urine and feces excretion of uranium were measured by Inductively Coupled Plasma—Mass Spectrometry (ICP-MS) analysis to determine the biokinetics of uranium (Figs. 1 and 2).

The initial dose incorporated by mice after the first instillation was measured 1 h after exposure by collecting tissues from the kidneys, lungs, gastrointestinal tracts, nasal compartment, and remaining carcasses (Fig. 2a). High uranium retention was recorded in the nasal compartment (NC, 4624 ng/g of tissue), 20-fold more than in control animals (p < 0.001), whereas the lung compartment only retains 21 ng/g at this time (tenfold more than the control group, p < 0.001). Gastro-intestinal tract (GIT) content reaches 1388 ng/g but represents only 1.6-fold the control group uranium content. From 1 h after exposure, uranium reaches the kidneys with no difference between the right and left kidneys (77 vs 78 ng/g) at a level 13-fold higher than control animals (p < 0.001) (data not shown).

Overall, a dose-dependent accumulation (p < 0.001 between each animal group per dose) is observed with a double peak of tissue or excreta (urine, feces) content corresponding to the two weeks of uranium treatment for all the tissues (kidneys, lungs, bones, carcasses) analyzed for uranium content (Fig. 1).

The pulmonary biokinetics of uranium are consistent with the established ICRP model for inhalation^33^. Pulmonary passage is proportional to the dose administered by repeated instillations, but a smaller proportion reaches the lung than expected (Fig. 1a). Uranium accumulation peaks are observed at D4 and D11, 24 h after the first series of exposure and 24 h after the second series of exposure respectively. A significant uranium level is detected in the lungs of mice 91 days after first exposure for doses 0.25 and 3 mg/kg/day (p < 0.001, Control vs uranium exposed), although it decreases by a factor of 10 (p < 0.05) between day 11 and day 91 for mice exposed to the highest uranium concentration (3 mg/kg/day), and by a factor of 5 for the group exposed to 0.25 mg/kg/day.

Renal retention is also proportional to the uranium dose administered for doses between 0.03 and 0.25 mg/kg/day (Fig. 2b), whereas the level of uranium in the tissue increases exponentially (p < 0.001) for doses of 1 to 3 mg/kg/day (Fig. 1c). The renal retention of uranium is thus modified as a function of the dose administered (p < 0.001 between D2 and D4, D4 or D7 and D11, D21, D91), probably influenced by the nephrotoxicity of uranium. Two accumulation peaks (D4 and D11) are observed for concentrations up to 1 mg/kg/day whereas only one peak on day 4 is observed for animals exposed to 3 mg/kg/day. The decrease in uranium concentration between the two peaks of uranium accumulation is less marked for 1 mg/kg/day than for 0.25 mg/kg/day. Retention is observed over the longer term (D91) as shown on Fig. 1c: kidney uranium content remains around 0.1 µg/g at D91 for the lowest uranium exposed group (p < 0.001). The level of uranium for the group exposed to the highest dose also decreases 7-times from D4 to D91 (p < 0.001), but remains at around 10 µg/g of kidney weight (a renal concentration known to be generally nephrotoxic). The data acquired over several experimental procedures were grouped together in the same data set and normalized with respect to the uranium administered dose for the renal retention model shown in Fig. 2b. Renal retention is proportional to the dose administered up to 1 mg/kg/day with a good superposition of the normalized level and retention increases exponentially for the highest uranium dose. This confirms that the renal retention of uranium is probably altered at high doses due to renal impairment.

Uranium retention in bone and whole body (carcass) varies in proportion to the uranium dose administered. The first accumulation peak was observed on D4 and the second on D11 (Fig. 1b,d). Interestingly, bone retention of uranium on D91 is higher than in other tissues. For the uranium exposed groups, the bone uranium content is diminished between D11 and D91 by 3.8-fold (p < 0.001) for the group exposed to 3 mg/kg/day and threefold for the group exposed to 0.25 mg/kg/day (p < 0.001) respectively. The remaining carcass uranium level is similar to bone uranium content: uranium concentration increases significantly (p < 0.001) from D2 to D91 for mice exposed to 1–3 mg/kg/day compared to non-exposed animals whereas the augmentation is only significantly increased (p < 0.05 for D7, p < 0.001 for D2, D4, D11) from D2 to D11 for animals exposed to 0.25 mg/kg/day compared to non-exposed animals.

Finally, the excretion of uranium in the feces and urine reflects tissue content with an increase in the quantity of uranium during the 2 exposure periods. A significant increase in urinary level is observed from D2 to D91 for the group exposed to 3 mg/kg/day (p < 0.05), from D2 to D11 for the group exposed to 1 mg/kg/day and on D2, 4 and 11 for the lowest dose (p < 0.05).The fecal uranium level is significantly higher than the controls on D2, D4 and D11 for animals exposed to 1 mg/kg/day (p < 0.05) and 3 mg/kg/day (p < 0.001).

### Clinical follow-up

Body weight, urine volume, feces weight, and creatinine are monitored at each time point (D2, 4, 7, 11, 21 and 91) as shown in Table 1. Regular monitoring of the animals revealed time- and dose-dependent weight loss. Body weight does not vary significantly for the control group and the group exposed to 0.25 mg/kg/day between D0 and D91. Between D2 and D11, the body weight of animals exposed to 1 mg/kg/day decreased by up to 10% compared to control non-exposed mice (p < 0.01). The body weight loss is more pronounced (up to 16%) for those exposed to 3 mg/kg/day (p < 0.001) compared to unexposed mice and occurred also from D2 to D11, the 2 weeks of repeated exposure to uranium (Table 1). From D21, body weight is similar for all groups of exposed or non-exposed mice, which return to a non-pathological body weight.

**Table 1:** Body weight and weight gained or lost since day 1 of exposure to uranium.

Dose (mg/kg/day)							
Days post instillations	0	2	4	7	11	21	91
NE							
Average weight (g)	24.4 ± 1.5	24.1 ± 1.8	24.2 ± 1.4	24.6 ± 1.5	24.3 ± 1.2	23.7 ± 2.2	29.8 ± 2.1
% Gain/lost since day 0	100	98.5 ± 3.5	99.1 ± 1.6	100.7 ± 2.6	98.3 ± 4.3	98.0 ± 3.8	120.5 ± 4.9
Number (n)	28	23	11	15	8	12	8
0.25							
Average weight (g)	24.0 ± 1.2	23.6 ± 1.4	23.4 ± 1.1	23.9 ± 1.0	23.2 ± 1.1	24.6 ± 0.9	28.1 ± 1.8
% Gain/lost since day 0	100	98.1 ± 3.6	97.6 ± 1.9	98.4 ± 2.3	97.5 ± 2.3	97.8 ± 2.5	118.3 ± 6.6
Number (n)	28	24	11	15	8	12	8
1							
Average weight (g)	24.2 ± 1.4	22.7 ± 1.4	22.9 ± 0.5	22.9 ± 0.8	23.0 ± 0.7	23.1 ± 1.1	ND
% Gain/lost since day 0	100	93.9 ± 2.7 ***	90.4 ± 2.4**	94.4 ± 3.9***	90.3 ± 2.2***	100.3 ± 4.2	
Number (n)	20	16	4	8	4	4	
3							
Average weight (g)	24.3 ± 1.5	21.7 ± 1.6	20.2 ± 1.5	20.9 ± 2.3	21.1 ± 1.9	23.8 ± 1.7	27.9 ± 2.5
% Gain/lost since day 0	100	88.7 ± 3.5***	83.6 ± 2.4***	86.7 ± 3.6***	87.0 ± 4.6***	93.5 ± 1.2	115.0 ± 7.7
Number (n)	28	24	12	16	8	12	8

The percentage of weight gain or loss is calculated for each animal and is related to its initial weight at D0. The number of animals decreases overall over time due to the successive animals’ euthanasia apart from the occasional absence of weighting due to technical constraints. *NE* non-exposed, uranium doses = 0.25, 1 or 3 mg/kg/day. Days = Time in days since the first day of exposure to uranium. *p < 0.05/**p < 0.01/***p < 0.001, comparison with non-exposed animals, Two-way ANOVA.

Diuresis is increased almost 4 times in animals treated with 3 mg/kg/day compared to unexposed animals on D7 (p < 0.01) while the amount of feces decreases 2 to 5 times between D4 and D11 for the same dose of uranium (p < 0.05) (Table 2). The amounts of diuresis and feces content do not differ for animals exposed to 1 mg/kg/day or lower compared to the control group. Glomerular filtration rate (GFR) does not vary significantly as a function of uranium dose. Nevertheless, a significant increase in GFR (threefold, p < 0.05) is observed between D4 and D7 for animals exposed to 3 mg/kg/day of uranium.

**Table 2:** Measurement of urine volumes, feces and glomerular filtration rate after uranium exposure.

Dose (mg/kg/day)						
Days post instillations	2	4	7	11	21	91
NE						
Diuresis (mL)	0.83 ± 0.36	0.84 ± 0.29	0.83 ± 0.22	0.62 ± 0.35	0.68 ± 0.44	0.99 ± 0.55
GFR (mL/min/kg)	2.9E−6 ± 1.4E−6	2.9E−6 ± 1.0E−6	2.8E−6 ± 0.2E−6	2.6E−6 ± 1.5E−6	1.7E−6 ± 1.1E−6	2.1E−6 ± 0.7E−6
Feces (g)	1.07 ± 0.27	1.15 ± 0.33	1.29 ± 0.15	0.94 ± 0.23	0.90 ± 0.38	0.75 ± 0.36
Number (n)	4	4	4	3	4	8
0.25						
Diuresis (mL)	0.59 ± 0.36	0.87 ± 0.17	0.98 ± 0.16	0.74 ± 0.30	0.58 ± 0.26	0.83 ± 0.28
GFR (mL/min/kg)	2.4E−6 ± 0.8E−6	3.9E−6 ± 1.4E−6	2.9E−6 ± 0.7E−6	3.7E−6 ± 2.1E−6	2.4E−6 ± 0.9E−6	2.8E−6 ± 0.7E−6
Feces (g)	1.13 ± 0.28	1.23 ± 0.26	1.29 ± 0.05	1.15 ± 0.18	1.23 ± 0.16	0.49 ± 0.18
Number (n)	4	4	4	3	4	8
1						
Diuresis (mL)	0.63 ± 0.25	0.76 ± 0.27	1.11 ± 0.49	0.51 ± 0.38	0.65 ± 0.23	ND
GFR (mL/min/kg)	2.4E−6 ± 0.4E−6	2.9E−6 ± 1.7E−6	2.0E−6 ± 1.4E−6	1.2E−6 ± 0.4E−6	2.6E−6 ± 0.6E−6	
Feces (g)	1.09 ± 0.22	0.73 ± 0.54	1.14 ± 0.26	0.87 ± 0.19	1.22 ± 0.35	ND
Number (n)	4	4	4	4	4	
3						
Diuresis (mL)	0.53 ± 0.50	0.96 ± 0.70	1.95 ± 0.57 **	1.88 ± 1.41	1.03 ± 0.49	1.39 ± 0.81
GFR (mL/min/kg)	0.9E−6 ± 0.8E−6	0.8E−6 ± 0.4E−6	2.6E−6 ± 2.0E−6	1.1E−6 ± 0.3E−6	2.4E−6 ± 0.7E−6	3.7E−6 ± 2.3E−6
Feces (g)	0.36 ± 0.42 *	0.17 ± 0.05 *	0.61 ± 0.33 **	0.26 ± 0.18 **	1.17 ± 0.21	0.74 ± 0.29
Number (n)	4	4	4	4	4	8

*NE* non-exposed, *GFR* glomerular filtration rate

*P < 0.05/**P < 0.01/***P < 0.001, comparison with unexposed animals, Two-way ANOVA.

### Doppler-ultrasonography (Doppler-US)

**Figure 3:**
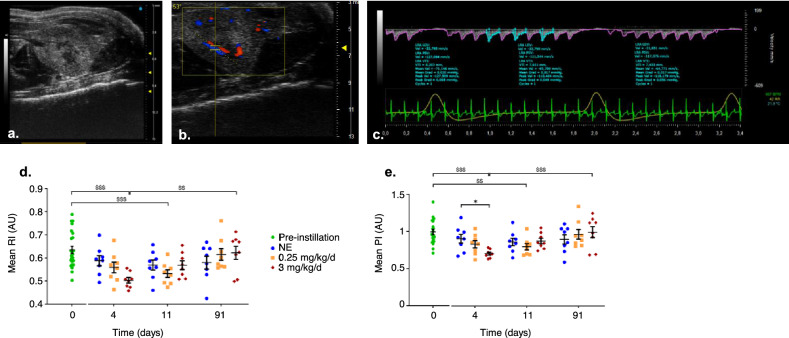
Renal morphological and functional follow-up by ultrasound. A-C: Representative ultrasound images of the left kidney in mice exposed to uranium at 3 mg/kg/day for 91 days. B-mode (**a**), Color-mode (**b**) and corresponding Pulse-Wave doppler of the intrarenal artery (**c**) on Vevo-Lab® software (V5.6.1). D-E: Resistive index (RI) (**d**) and Pulsatile index (PI) (**e**) levels of intrarenal arteries obtained for mice before exposure (green), during exposure (D4), just after exposure (D11) and 3 months after exposure (D91) at 0.25 or 3 mg/kg/day. Each point represents an independent animal. The pre-instillation group (D0) includes all animals that were monitored for 4, 11 or 91 days after the first instillation to uranium or the vehicle solution. The non-exposed group (NE) received the vehicle solution (sodium bicarbonate) at the same time point as uranium exposed animals. n = 8 for each time and dose. The values are expressed as mean ± SEM. *P < 0.05/**P < 0.01/***P < 0.001, comparison with unexposed animals, Holm-Sidak test.

Mice underwent a Doppler-US examination on D0, D4, D11 and D91 to evaluate morphological and functional changes to the kidneys. During the follow-up by Doppler-US, morphological changes were assessed: the left and right kidneys were screened for any change in kidney tissue echogenicity or kidney length (Fig. 3a). The kidneys did not show any visible morphological changes nor visible lesions (hypoechoic cysts or hyperechoic fibrotic scars) regardless of the analysis period. Intrarenal arteries are visible in color mode and vascular parameters are obtained from at least 3 different peaks from the expiration phase with a heart rate of 400–500 BPM (Fig. 3b,c) Vascular parameters are monitored and the PI and RI calculated (Fig. 3d,e respectively) are similarly altered as a function of uranium exposure dose and time. A time-dependent change is observed independently of the dose for PI and RI levels (not shown): PI and RI are significantly altered (p < 0.001) between D0 (before exposure) and D4 or D11 after the beginning of the experiment (corresponding to the final days of exposure on each week). Moreover, dose-dependent changes are apparent on D4: PI decreased by 30% (p < 0.001) for the highest dose (3 mg/kg/day) compared to the control non-exposed group and a return to physiological level is observed on D91. Similarly, the lower dose (0.25 mg/kg/day) induces a decrease of 20% (p < 0.01) on D11 compared to D0 and a return to physiological values on D91. This could highlight the cumulative effect of repeated exposure to uranium: 8 times exposure to 0.25 mg/kg/day results in a slight change in PI and RI on D11, whereas 4 doses of 3 mg/kg/day induce greater changes in PI and RI, as early as D4.

### Renal anatomopathology

**Figure 4:**
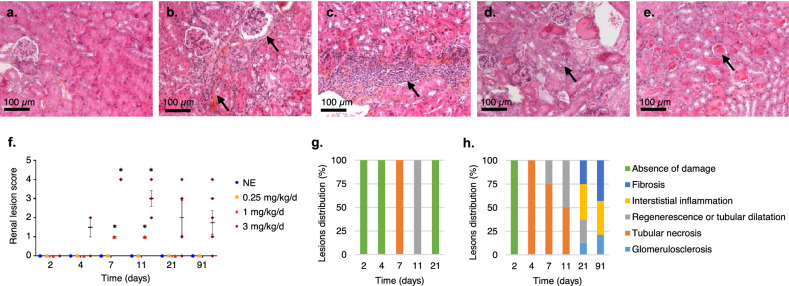
Assessment of renal damage by histopathological examination after exposure to uranium (0.25–3 mg/kg/day) by intranasal instillation. (**a**–**e**) Kidney longitudinal section microphotographs (200×) representative of damage observed after HES staining, scale bar = 100 µm. (**a**) Normal kidney. (**b**) Glomerulosclerosis and interstitial fibrosis. (**c**) Interstitial inflammation. (**d**) Tubular regeneration. (**e**) Tubular necrosis. (**f**) Total damage score (all criteria combined) according to exposure dose and time since first instillation. Each point represents an independent animal. (**g**,**h**) Percentage distribution of the different impairments for animals exposed to 1 mg/kg/day (**g**) or 3 mg/kg/day (**h**). The values are expressed as mean ± SD, n = 3–4 for each time and dose. *P < 0.05, comparison with unexposed animals, Two-way ANOVA.

Representative microphotographs of renal lesions induced by repeated uranium exposure in mice are shown in Fig. 4a–e. Global scoring of the damage induced in the kidney, and the extent of this damage were quantified. No lesions were observed for animals exposed to the lowest uranium concentration and control groups over any observation period. Seven and eleven days after the first treatment at 1 mg/kg/day, global damage had significantly increased in mice kidneys compared to control animals (Fig. 4f), resulting in the transient appearance of tubular necrosis and tubular regeneration/dilatation respectively (Fig. 4g). Finally, the total damage score is higher (2–threefold) for the 3 mg/kg/day group. Significant structural damage was observed on D7, D11 and D91 (p < 0.05) and non-significant damage on D4 (p = 0.06) and D21 (p = 0.07) (Fig. 4f). In the short term after first exposure, necrosis and tubular dilatation were induced while beyond D21, glomerulosclerosis, fibrosis and interstitial inflammation were observed (Fig. 4h). The extent and type of renal lesions induced by uranium is dose and time dependent.

### Urinary and renal protein KIM-1 levels

**Figure 5:**
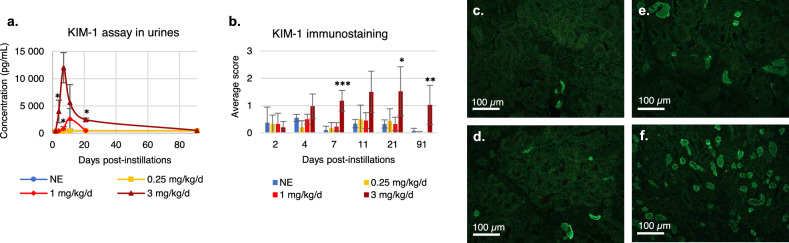
Urine assay and immunostaining for KIM-1 after 0.25–3 mg/kg/day uranium exposure. Urine is collected by passing through a metabolic cage 16 h before the euthanasia of the animals. The kidneys are collected after the euthanasia. KIM-1 expression is measured in urine using ELISA (**a**) and by immunohistochemistry on a longitudinal kidney section (**b**–**f**). (**b**) Mean score over 10 fields according to exposure dose and time since first instillation. (**e**,**f**) Representative microphotographs corresponding to each score from 0 to 4, scale bar = 100 µm. *NE* non-exposed group. The values are expressed as mean ± SD, n = 3–4 for each time and dose. *P < 0.05/**P < 0.01/***P < 0.001, comparison with unexposed animals, Two-way ANOVA.

KIM-1, a known damage marker for proximal convoluted tubules, was measured in kidneys and in urine using different methods. ELISA urine testing shows no variation over time for control animals or exposed to 0.25 mg/kg/day of uranium. A significant increase in KIM-1 level was observed on D7 (2.5-fold, p < 0.05) and a non-significant increase on D11 (sevenfold, p = 0.057) for the 1 mg/kg/day group (Fig. 5a). For the 3 mg/kg/day animals, a significant tenfold increase occurred on D4 (p < 0.05), a peak was recorded on D7 (30-fold, p = 0.057) followed by the beginning of decrease in level which remained significant on D21 (sixfold, p < 0.05). KIM-1 content in renal tissue was measured by IHC on longitudinal kidney sections. Ten images per section were scored by semi-quantification from 0 to 4 according to the number of tubules labelled, the extent and the intensity of staining (Fig. 5c–f). For animals exposed to uranium doses lower than 1 mg/kg/day, the evaluated tubules were marked at 0 or 1. This immunostaining showed a persistent increase in KIM-1 expression from D7 to D91 for animals exposed to 3 mg/kg/day of uranium compared to mice exposed to lower doses (Fig. 5b).

### Renal gene expression of nephrotoxicity markers

**Figure 6:**
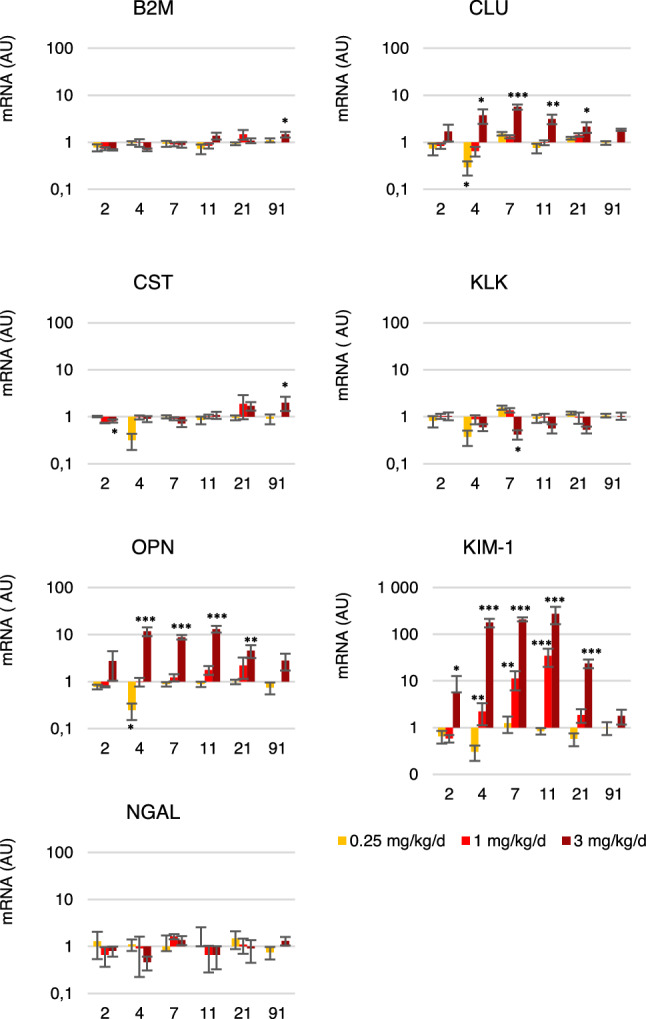
Gene expression of nephrotoxicity biomarkers in the renal tissue after uranium exposure (0.25–3 mg/kg/day). Results are expressed as a ratio to the expression of the housekeeping gene HPRT. *AU* arbitrary unit, *B2M* β-2 microglobulin, *CLU* clusterin, *CST* cystatin, *KIM-1* kidney injury molecule 1, *KLK* kallikrein, *NGAL* lipocalin 2, *OPN* osteopontin. The values are expressed as mean ± SD, n = 3–4 for each time and dose. *P < 0.05/**P < 0.01/***P < 0.001, comparison to unexposed animals, Two-way ANOVA.

Exposure to uranium significantly altered the expression of KIM-1 over time (p < 0.05) for doses higher than 1 mg/kg/day between D2 and D4 (Fig. 6). Similarly, from D11 to D91, KIM-1 mRNA level returned to a basal level of expression. The dose-dependent effect is noticeable from D2, for animals exposed to the highest dose (5 to 275-fold, p < 0.05), and from D4 to D11 for the 1 mg/kg/day dose (2 to 34-fold, p < 0.01). No significant differences were observed for lipocalin-2 (NGAL), a very early diagnostic marker of proximal convoluted tubule injury, regardless of exposure dose or time. Osteopontin (OPN) is naturally expressed in renal tissue but may increase in case of glomerulonephritis or tubulointerstitial nephritis. A significant decrease (threefold, p < 0.05) in its expression was recorded on D4 for animals exposed to 0.25 mg/kg/day of uranium. A time-dependent effect was noticeable for the highest dose between D11 and D91 (p < 0.05). Between D4 and 21, a significant increase in the expression of OPN compared with unexposed animals was detected in mice treated with 3 mg/kg/day (p < 0.01). Conversely, a decrease equal to half of the kallikrein (KLK) expression, a marker that decreases in case of renal injury caused by a nephrotoxic agent, was visible at D7 for this same dose. Its expression decreased significantly between D2 and D7 and then returned to the basal expression level from D7 to D91 (p < 0.05). After exposure to 3 mg/kg/day of uranium, β2 microglobulin (B2M) and cystatin C (CST) expression ware doubled on D91 compared to unexposed animals (p < 0.05). Both markers increase in case of tubular and glomerular dysfunction or damage. Clusterin (CLU) is an early marker of acute tubular damage, whose expression was decreased (threefold) after treatment with 0.25 mg/kg/day of uranium on D4, whereas it was increased (2 to fivefold) from D4 to D21 with a peak of expression on D7 for the 3 mg/kg/day dose. The time effect was observed from D7 to D91 for this same dose (p < 0.01).

## Discussion

The kidney and more specifically the proximal convoluted tubules are the primary site of uranium accumulation^34^, but knowledge of the nephrotoxicity threshold is inadequate depending on the route and mode of exposure, particularly after inhalation^35^. The objectives of our study were to experimentally investigate the biokinetics of uranium after repeated exposure via the upper airway in mice and determine a nephrotoxicity threshold and then test whether this threshold affects uranium retention and excretion. Then we experimentally modeled exposure to uranium via the upper airways, that can concern nuclear cycle workers. It addresses, for the first time, the monitoring of uranium accumulation as a function of time and dose during and after exposure period to uranium. The biokinetic modeling of uranium under these conditions of exposure by repeated instillation (exposure via the upper respiratory tract) in mice was established from published models^25,33,36^ and adjusted based on the exposure protocol: repeated acute exposure by intranasal-instillation^23^.

The retention of uranium in the kidneys, lungs, bones, and carcass showed a double peak of accumulation as a function of time, corresponding to the end of each week of instillation (Fig. 1). There is a linear relationship between exposure dose and body accumulation for doses below 1 mg/kg/day, while it accumulates exponentially between doses of 1 and 3 mg/kg/day. Previous works on repeated exposure via the upper airways studied biokinetics at the end of the treatment period without following the critical period of repeated exposure^27,37^. Thus, we showed that the renal biokinetics covered by our model are similar to a sum of acute subcutaneous or intraperitoneal exposures to 2 mg/kg uranium in rats^38,39^ or to repeated exposure of rats by inhalation of UO_2_^27^. Unlike exposure by intratracheal instillation^40,41^, intranasal instillation makes it possible to more closely mimic exposure by inhalation, as it also includes the upper airways (extra-thoracic compartment) and the digestive tract. One hour after exposure by intranasal instillation, uranium is mainly found in the gastro-intestinal tract, the extra-thoracic compartment, and the lung (Fig. 2a). Uranium also reaches the kidneys without any differences between the right and left kidneys (data not shown). Exposure by instillation offers the advantages of being reproducible, the dose administered is controllable, and it can be used to treat a large number of animals at the same time. This exposure model has been previously validated for uranium^32^ and aluminum exposure^30^. The elimination time of uranium is very similar to that described during the inhalation of uranium, namely, rapid elimination by urine and feces in the first days after stopping treatment and significantly slower elimination after this point^27,33,37^.

Uranium lung retention is consistent with the model established by the ICRP and proportional to the dose administered^23^, although a lower than expected dose reached the lung. The renal retention of uranium is also proportional if the dose administered is less than 1 mg/kg/day, while uranium renal accumulation increases exponentially for a concentration above 1 mg/kg/day. For the highest exposure dose, the renal accumulation peak was observed on D4, whereas it was observed on D11 for lower doses (Fig. 1). After chronic exposure to 0.3 g of uranium (35 µg/g of kidney) via a gastrocnemius implant in rats, it takes 3 months to reach 50% of this concentration in the organ^42^. In case of chronic exposure through drinking water at 600 mg/L, a renal accumulation 10 times lower is found after 9 months of exposure^12^. On D21, i.e. 10 days after the end of exposure to 1 mg/kg/day, the renal retention reaches 5 µg of uranium/g of kidney, a value close to that of^43^ for acute intramuscular exposure of rats to 1 mg/kg of uranium or^39^ for acute exposure of mice to 2 mg/kg intraperitoneally. Thus, the elimination kinetics obtained during this study differ from those of chronic exposure, but are similar to repeated acute contamination. However, the renal retention of uranium is modified for high concentrations during repeated exposure (> 1 mg/kg/day) probably due to the functional renal impairment induced by this uranium exposure.

Kidney damage is the first symptom of uranium poisoning^8,34^. The risk can be better assessed using the relationship between the concentration in the organ and the harmful effects observed. In fact, in the event of tubular and glomerular damage, functional and morphological analyses are needed. Ultrasound, as well as urinary, tissue and plasma diagnostic markers, such as the measurement of creatinine or the KIM-1 assay, CLU and OPN are good sensitive markers used in humans and animals, and representative of histological damage that can be altered by uranium^2,7^. Short-term tubular damage (dilatation and necrosis) from the end of the first week of instillation for the 3 mg/kg/day dose (i.e. a uranium content in the kidneys of 72 µg/g) and from D7 for the 1 mg/kg/day (5.6 µg/g of uranium in the kidneys) are actually observed according to the histological analysis (Figs. 1 and 4). The onset of this morphological damage corresponds to peak uranium accumulation in the kidneys observed for each of the doses (Fig. 1). Similar tubular damage was observed in kidneys greater than 10 µg/g after a single administration of uranium in rats by subcutaneous injection^44^, greater than 8.2 µg/g after intraperitoneal injection^39^ or greater than 22 µg/g after intramuscular injection^43^. Tissue regeneration from D7 for the highest dose (Fig. 4) can be observed by monitoring over time up to 91 days after exposure by intranasal instillation, whereas it is necessary to wait 28 days after a single intraperitoneal administration of 4 mg/kg in Swiss mice^39^ or 15 days after 10 mg/kg administered subcutaneously in rats^5^. On D91, the integrity of the renal tissue appears to be globally restored, but inflammation and fibrosis persist for the dose of 3 mg/kg/day (10 µg of uranium /g of kidney), resembling long-term renal damage (Figs. 1 and 4)^5^. The appearance of dilated tubules and necrosis is mainly influenced by the dose and mode of exposure (acute/repeated VS chronic) to uranium, while regeneration is mainly influenced by time and route of exposure. Nevertheless, we failed to observe any major morphological changes in renal ultrasound monitoring (Fig. 3). The renal ultrasound study was previously completed during a single study of exposure to uranium in rats (intraperitoneal administration of 5 mg/kg of uranium)^45^. The authors describe the appearance of fibrotic zones containing atrophied or dilated tubules but after exposure to a higher single dose of uranium, and results were not correlated with histological analyses, which makes comparison difficult.

Tubular and glomerular morphological damage was also assessed by in-situ gene expression analysis. The overexpression of inflammatory markers (OPN) and tubular damage (CLU and KIM-1)^46^ were observed from D4 to D21 for the highest exposure dose (Fig. 6). At the lower dose (1 mg/kg/day) only KIM-1 increased from D4 to D11, i.e. for uranium content in kidneys greater than 5.5 µg/g. KIM-1 is indeed a very sensitive marker and can be used to evaluate renal damage. It has previously been proven that a link exists between overexpression of KIM-1 and uranium-induced renal failure^22,47^. Inflammation is a uranium toxicity mechanism involving the recruitment of inflammatory cells^4,48^. KIM-1, CLU and OPN are increased after chronic exposure to uranium through drinking water followed by treatment with gentamicin^49^ or after a single intraperitoneal injection of uranium^4,50^. Repeated exposure to uranium by intranasal instillation at doses less than or equal to 1 mg/kg/day induces morphological damage similar to transient acute renal failure, whereas the highest dose induces acute damage followed by chronic renal failure, in particular because of the persistent inflammation it induces (Figs. 4h and 6).

The functional consequences of this renal morphological damage induced by uranium are evaluated by clinical biochemistry assays, measuring the urinary excretion of KIM-1, and thanks to long-term longitudinal monitoring (3 to 91 days after exposure) by high-resolution Doppler-ultrasound. A decrease in GFR associated with an increase in urinary excretion of KIM-1 was observed on D4, i.e. at peak uranium accumulation in kidneys for animals exposed to 3 mg/kg/day (Table 2 and Fig. 5). Decreasing GFR is a classic clinical parameter indicating a significant loss of function, particularly at the level of the glomeruli. Urinary excretion of KIM-1 shows functional impairment of the proximal convoluted tubules, the main site of uranium accumulation in the kidneys^34,46^. Renal blood supply represents 25% of cardiac output and it can be quantified by measuring the renal vascular resistance indices by Doppler ultrasound (Fig. 3). No previous work deals with the study of renal blood flows in the context of radiochemical exposure. The Doppler analysis carried out in our study makes it possible to detect renal functional impairment over time after uranium exposure (< 3 mg/kg/day). The effect of uranium is transient since we observed changes in PI and RI between D4 and D11 then a return to a basal state on D91, probably correlating with the tubular regeneration observed in histopathology. Interestingly, our data showed a reduction in PI & RI in the kidneys, indicating a reduction in renal blood flow resistance. These results assume a slight functional impairment from a dose of 0.25 mg/kg/day without morphological impairment as described earlier. A reduction in RI can be attributed to pre-glomerular vasodilation mediated by myogenic vasoconstriction^51^, part of the autoregulation of the renal system. Other studies in humans have associated increased RI (resistive index) with shock (cardiogenic, hypovolemic, septic, etc.)^52^ or persistent acute renal injury (AKI)^53^. In mice, chronic pathologies such as diabetes^54,55^ or persistent AKI induced by a nephrotoxic agent such as cisplatin^56^ are associated with an increase in RI in these studies. The latter authors also noted that the RI does not vary in patients with transient AKI. By contrast^45^, described a decrease in RBF (renal blood flow) in the presence of uranium, but the latter is not quantified in their study. A reduction in RBF reflects reduced glomerular filtration capacity due to less efficient blood perfusion.

## Conclusion

Repeated intranasal instillation of uranium is a biokinetic model that mimics exposure through the upper airways. The morphological and functional renal damage observed from exposure to a 1 mg/kg/day dose therefore means that the nephrotoxicity threshold of our exposure model is between 0.25 and 1 mg/kg/day, which is similar to the thresholds described for other routes of acute uranium exposure. The adapted biokinetic model of acute exposure is consistent with the data obtained in mice after repeated exposure. We also show that this model is no longer valid for exposure to a highly nephrotoxic dose due to the induced renal failure.

Thanks to this work, we have shown that our experimental model could be used in other studies to mimic occupational exposure to uranium. Indeed, recent epidemiological studies show an excess risk of the development of renal cancer for nuclear fuel workers exposed to uranium^57,58^ and raise the question of the potential link between renal cancer and uranium exposure^59,60^. This question could be answered using an experimental model of exposure like the one we developed in this study with a good knowledge of uranium biokinetics and nephrotoxicity thresholds.

## Materials and methods

### Animals

Experiments were performed on 8-week year old male C57BL/6 J mice provided by Charles River (France). Animals were housed at a constant room temperature (21 °C ± 1) with a 12 h:12 h light–dark cycle. Water and food were supplied ad libitum. Body weight, urine volume and feces were monitored at regular time points from the first day of exposure to the last time point.

### Animal uranium exposure

Mice were subdivided into groups of 4 animals per dose per time until D21, and into groups of 8 animals for D91. Animals in the contaminated group were exposed to uranyl nitrate (UO_2_(NO_3_)_2_; U238: 99.74%, U235: 0.26%, U234: 0.001%, Merck-Prolabo) dissolved in 100 mM of sodium bicarbonate at different uranium solution concentrations to deliver the desire amount of uranium to mice (0.03, 0.125, 0.25, 1 or 3 mg/kg/day) with the same volume administered (15 µl). Control animals were instilled with a solution of 100 mM of sodium bicarbonate. The animals were instilled once a day for 4 days, followed by a 3-day break and then another 4 days of contamination by intranasal-instillation of 15 µL as previously described^32^.

### Small animal functional imaging

The VEVO 3100 High resolution Ultrasound (US) imaging system (Fujifilm Visualsonic Inc) with the MX550D probe (40 MHz) was used to acquire kidney sagittal images and Doppler measurements. Eight-week old C57BL/6 J mice were monitored by renal echography before (D0) and after uranyl nitrate instillation, at regular time points (D4, D7, D21 and D91). A US examination was performed on both animal kidneys. All animals were anaesthetized with isoflurane (induction 2%; maintenance: 0.75–1% to keep heart rate above 400 BPM) (Aerrane) and held on a platform heated to 37 °C (Fujifilm Visualsonic Inc), designed to monitor physiological parameters (ECG and respiration rate). The animals' abdomens were then depilated with a shaver and depilatory cream (Cosmia). An ultrasound transmission gel (Centravet) is applied to clean skin. B-mode images were acquired of the kidney to detect any morphological alteration to the whole kidney using the three-dimensional motor to scan the left and right kidneys. Images were recorded every 0.76 mm to reconstitute a three-dimensional image of the kidney. PW-Doppler images were acquired for the intra-renal arteries for the left and right kidneys at angles between 41 and 53°, PRF was fixed at 20 kHz, the transducer (MX550D) was positioned to acquire sagittal sections of the kidneys. All data acquired were then analyzed using Visualsonic’s Vevolab software: for each animal and at each time-point, both kidneys' arterial fluxes were analyzed with three different peaks to acquire intra-renal flow velocity. Renal pulsatile index and resistive index (PI and RI respectively) were automatically determined by the Vevolab® software using the following constructor formulas: PI = (PSV-EDV)/MV, RI = (PSV-EDV)/PSV; where PSV = Peak Systolic Velocity, EDV = End Diastolic Velocity and MV = Velocity Time Integral (VTI) Mean Velocity.

### Urine and feces collection

During each collection period, animals were placed individually in standard metabolic cages (Techniplast) for a 16 h period to collect urine and feces samples. Urine was centrifuged at 3000×*g* for 10 min and supernatants were isolated and stored at −80 °C.

### Plasma and tissue collection

At each time point, mice were euthanized by terminal exsanguination (intracardiac puncture) and cervical dislocation under isoflurane anesthesia. Both kidneys were collected and sagitally cut: half of the left kidney was placed in formaldehyde 4% for preservation for 24 h, the other half of a kidney was flash-frozen in liquid-nitrogen and stored at − 80 °C. The right kidney, both lungs, back legs bones, nasal compartment, gastrointestinal tracts and carcass were stored at − 20 °C for quantification by Inductively-Coupled Plasma—Mass Spectrometry (ICP-MS). Blood samples were centrifuged at 3000×*g* for 10 min to obtain plasma which was then stored at − 80 °C.

### Glomerular filtration rate (GFR)

Plasma creatinine was measured with the fluorometric Creatinine assay kit (ab65340, Abcam), and urine creatinine with the colorimetric Creatinine assay kit (ab204537, Abcam).

### Uranium biokinetics

The level of uranium was quantified by ICP-MS (iCAP Q, Thermo Fisher Scientific) in the kidneys, lungs, bone femurs, urine, feces, gastrointestinal tracts, nasal compartment, and carcasses. Beforehand, carcasses and feces were ashed at 500 °C. The organs were mineralized in nitric acid 69% and hydrogen peroxide 30%. The organs were mineralized using a microwave digestion furnace (Ethos One®, Milestone). The samples were then evaporated until dry and dissolved in nitric acid 20%. After appropriate dilution, uranium was quantified with bismuth as an internal standard and a uranium external calibration curve. The detection limit of uranium was determined by ICP-MS: 0.5 ng/L for ^238^U and 0.01 ng/L for ^235^U.

### Renal biodosimetric model

A specific biodosimetric model, SAAM II (Simulation, analysis, and modeling software for tracer and pharmacokinetic studies), was developed from published ones in order to model the data: the pulmonary model is based on the ICRP model for inhalation^33^, the gastro-intestinal tract model is derived from^25^ and the systemic model was determined by Leggett and Pellmar^36^. The fraction deposited in the nasal compartment was adjusted from observed pulmonary retentions to implement the specific deposition pattern corresponding to intra-nasal instillations.

### Histopathology

The half kidney preserved in 4% paraformaldehyde was dehydrated before being embedded in paraffin and cut with a microtome (5 µm section), stained with hematoxylin, eosin and saffron, and examined under brightfield microscope. Damage was assessed blindly by an external expert pathology laboratory (Vebio) according to standard criteria^61^. Glomerular damage was estimated using glomerulosclerosis and glomerular cystic dilatation. Tubule-interstitial damage was estimated based on tubule necrosis, regeneration and dilatation, and interstitial inflammation and fibrosis. The different kinds of lesions were scored from 0 to 4 for each animal (0: no damage/1: slight/2: moderate/3: marked/4: severe). The total sum of all lesions corresponds to the global lesion scoring. The percentage of lesion distribution represents the scope of the different types of impairment in relation to the total score.

### Immunostaining

Paraffin-embedded slices were deparaffinized and hydrated in descending gradations of ethanol and in 3% H_2_O_2_ to block endogenous peroxidase activity. Antigen retrieval was achieved with a pH6 citrate buffer. Sections were incubated overnight with anti-KIM-1 (Ab47635, Abcam) diluted to 1:200. After washing, slices were incubated with an Alexa-488 secondary antibody (ab150061, Abcam) diluted to 1:1000 and assembled with mounting medium (Vectashield, VWR). Ten microphotographs per animal were collected with a fluorescence microscope (Zeiss AxioImager). The fluorescence intensity of each image was manually scored from 0 to 4, depending on the number and area of labeled tubules.

### Real time RT-PCR

Total RNA was extracted from 20 to 30 mg of renal tissue using the RNeasy total RNA isolation kit (74106, Qiagen) and reverse-transcribed into cDNA using the High-capacity cDNA reverse transcription kit (4368814, Thermo Fisher Scientific). Real-time PCR was used to analyze the mRNA level of nephrotoxicity biomarkers : B2M (CACTGACCGGCCTGTATGCT/GGTGGGTGGCGTGAGTATACTT), CLU (TCGGGCATCTGGCATCA/AAGCTCACGGGCGAAGAAC), KLK (GCCCAACACCGGCTTGT/TGCTCATTCAGGAGGCTCATG), CST (GCGTTGGACTTCGCTGTGA/GGCTGTGGTACGCATCGTT), OPN (CCCTCGATGTCATCCCTGTT/TTCCGTTGTTGTCCTGATCAGA), KIM-1 (TTTCAGGCCTCATACTGCTTCTC/TGACCCACCACCCCCTTT), NGAL (CGGGACCTGGTACCTCGAT/ CCATTTTCTTCAATGCGAGTCA). Samples were prepared at a final concentration of 1 ng/µL cDNA per well. A mix containing 2.5% v/v primers (Fisher Scientific), 83% v/v SYBR (4367659, Thermo Fisher Scientific) and 14.5% v/v sterile water to yield a final volume of 10 µL was used. Samples were normalized to hypoxantine-guanine phosphoribosyl-transferase (HPRT) and fold induction calculated relative to the unexposed controls.

### KIM-1 assay in urine

KIM-1 was measured in urine using an ELISA kit according to the manufacturer’s instructions (DY1817, R&D). Urine was diluted to a concentration 1:10 to 1:50 to comply with the concentration intervals required for the assay.

### Statistical analysis

To compare the nephrotoxicity induced by different doses of uranium exposure, statistical analysis used two-way analysis of variance (ANOVA), in case of absence of normality Wilcoxon signed-rank testing, or Holm-Sidak testing had been used with uranium exposure and time as the two factors (Prism, R studio, Sigma plot). The level of signification was set to 0.05. The n value was specified in the legends of each figure.

### Approval for animal experiments

The Animal Care Committee #81 C2EA-IRSN of the Institute approved the experiments under the reference APAFIS#16305-2018072616221896 v2 delivered on December 13th, 2018, which were conducted in accordance with French regulations on animal testing (Ministry of Agriculture Act No. 2001-464, May 2001) and which complied with the ARRIVE guidelines.

## Data Availability

The datasets used and/or analysed during the current study available from the corresponding author on reasonable request.

## Abbreviations

B2M: Beta-2 microglobulin
BPM: Beat per minute
CLU: Clusterin
CST: Cystatin
GFR: Glomerular filtration rate
ICRP: International Commission on Radiological Protection
ICP-MS: Inductively-coupled plasma-mass spectrometry
KIM-1: Kidney injury molecule 1
KLK: Kallikrein
NGAL: Lipocalin 2
OPN: Osteopontin
PI: Pulsatile Index
RI: Resistive Index
US: Ultrasonography
